# Detection of maxillary sinus pathologies using deep learning algorithms

**DOI:** 10.1007/s00405-025-09451-4

**Published:** 2025-05-20

**Authors:** Ceren Aktuna Belgin, Aida Kurbanova, Seçil Aksoy, Nurullah Akkaya, Kaan Orhan

**Affiliations:** 1 Faculty of Dentistry, Department of Dentomaxillofacial Radiology, Hatay Mustafa Kemal University, Hatay, Turkey; 2 Faculty of Dentistry, Department of Dentomaxillofacial Radiology, Near East University, Mersin10, 99138 Turkey; 3 Dentmetria Inc, İstanbul, Turkey; 4 Faculty of Dentistry, Department of Dentomaxillofacial Radiology, Ankara University, Ankara, Turkey; 5 Medical Design Application, and Research Center (MEDITAM), Ankara University, Ankara, Turkey; 6 Faculty of Dentistry, Department of Oral Diagnostics, Semmelweis University, Budapest, Hungary

**Keywords:** Artificial intelligence, Deep learning, Cone beam computed tomography, Dentistry, Maxillary sinus, Convolutional neural network

## Abstract

**Purpose:**

Deep learning, a subset of machine learning, is widely utilized in medical applications. Identifying maxillary sinus pathologies before surgical interventions is crucial for ensuring successful treatment outcomes. Cone beam computed tomography (CBCT) is commonly employed for maxillary sinus evaluations due to its high resolution and lower radiation exposure. This study aims to assess the accuracy of artificial intelligence (AI) algorithms in detecting maxillary sinus pathologies from CBCT scans.

**Methods:**

A dataset comprising 1000 maxillary sinuses (MS) from 500 patients was analyzed using CBCT. Sinuses were categorized based on the presence or absence of pathology, followed by segmentation of the maxillary sinus. Manual segmentation masks were generated using the semiautomatic software ITK-SNAP, which served as a reference for comparison. A convolutional neural network (CNN)-based machine learning model was then implemented to automatically segment maxillary sinus pathologies from CBCT images. To evaluate segmentation accuracy, metrics such as the Dice similarity coefficient (DSC) and intersection over union (IoU) were utilized by comparing AI-generated results with human-generated segmentations.

**Results:**

The automated segmentation model achieved a Dice score of 0.923, a recall of 0.979, an IoU of 0.887, an F1 score of 0.970, and a precision of 0.963.

**Conclusion:**

This study successfully developed an AI-driven approach for segmenting maxillary sinus pathologies in CBCT images. The findings highlight the potential of this method for rapid and accurate clinical assessment of maxillary sinus conditions using CBCT imaging.

## Introduction

The maxillary sinus (MS) is one of the paranasal sinuses, located near the maxillary posterior teeth. Due to its proximity to dental structures, it is frequently affected by infections. It plays a critical role in various dentomaxillofacial surgical interventions, including apical surgeries and sinus augmentation for dental implant placement [1]. To ensure appropriate treatment planning, dentists must precisely determine the boundaries of the MS and assess the presence of any pathological conditions. Accurate diagnosis and classification of MS pathologies prior to surgical interventions are essential for achieving predictable clinical outcomes [2]. However, performing surgical procedures in patients with pathological changes in the MS may increase the risk of sinus infections [3].

Traditional two-dimensional radiographs have been used to evaluate MS conditions, but their effectiveness is limited due to overlapping anatomical structures, which can obscure pathology detection. Computed tomography (CT) has long been considered the gold standard for diagnosing sinus pathologies; however, its use is constrained by lower spatial resolution and a higher radiation dose. Cone beam computed tomography (CBCT) has emerged as a preferred alternative, offering higher-resolution images with reduced radiation exposure and without distortion and superimposition from adjacent structures [4, 5].

Although CBCT and CT are widely utilized for segmenting MS images, segmentation accuracy often depends on the clinician’s experience. Image artifacts and degraded quality can lead to misinterpretations, presenting a significant limitation. To overcome these challenges, artificial intelligence (AI)-assisted programs have been introduced for MS segmentation, demonstrating high accuracy and reliability [6, 7].

Machine learning, a subfield of AI, allows systems to recognize statistical patterns in datasets and make predictions without explicit programming [8]. Deep learning, a specialized branch of machine learning, is particularly effective in medical applications [9]. These algorithms rely on multilayer artificial neural networks [10] to extract hierarchical data representations, optimizing the learning process and reducing prediction errors. Convolutional neural networks (CNNs), a type of deep learning model, are frequently employed in image-related tasks such as automatic detection, segmentation, and classification of complex structures [6].

CNN-based models are widely utilized in medicine and dentistry. They have been successfully implemented in the detection and segmentation of organs, pathologies and metastases in organs [11–13]; in classification processes such as cancer stage classification and various histopathological classifications [14, 15]; and in research such as anatomical landmark detection [16]. In dentistry, CNNs have been applied in airway segmentation, mandibular canal detection [17, 18], fracture and pathology identification in the jaws [19, 20], and caries detection, as well as in dental treatment planning and implant assessments [21, 22].

This study aims to assess the accuracy of deep learning algorithms in detecting MS pathologies from CBCT images.

## Materials and methods

### Study design and patient selection

This study was conducted in accordance with the standards of the Helsinki Declaration on medical research. In this study, the plan and report followed the recommendations of Schwendicke et al. [23] for reporting artificial intelligence in dental research. The Noninterventional Clinical Research Ethical Committee of Near East University approved the study protocol (decision no. YDU/2024/128–1906). In our study, CBCT images of patients who were sent to the Dentomaxillofacial Radiology Department of Near East University for various reasons were scanned retrospectively. Patients who underwent surgery in the maxillary sinus region, who experienced trauma, who had tumors affecting the relevant region, or who had radiographs containing artifacts were excluded from the study.

### Dataset

A sample of 500 CBCT scans (1000 sinuses, 243 females and 257 males, mean age 50.82 years) from 2009 to 2016 with the same scanning parameters was collected. The scans were acquired via the same CBCT device (Newtom 3G (Quantitative Radiology s.r.l., Verona, İtalya)) with a field of view (FOV) of 23 × 17.3 cm and a 0.300 mm voxel size. The acquisition parameters were 120 kVp, 3–5 mA, 360° rotation and 36 s exposure time.

**Fig. 1:**
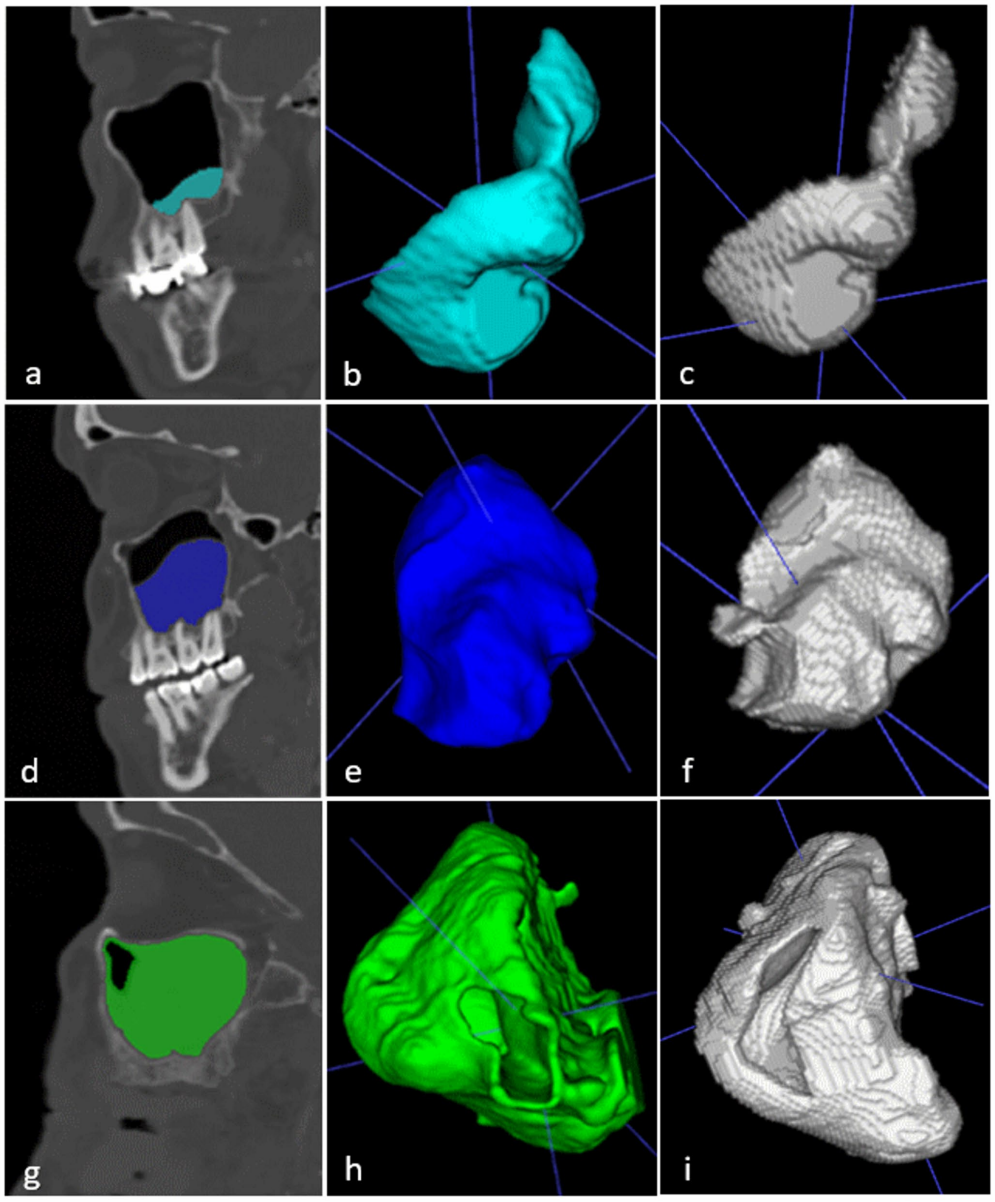
Manual and AI-based segmentation of maxillary sinus pathologies. (**a**–**c**) Mucosal thickening (**d**–**f**) Mucous retention cyst (**g**–**i**) Sinusitis. (**a**, **d**, **g**) sagittal slice manual segmentation, (**b**, **e**, **h**) 3D manual reconstruction, (**c**, **f**, **i**) AI-based 3D segmentation

The patient data were converted to Digital Imaging and Communication in Medicine (DICOM) files, and the CBCT images were exported anonymously. When any of the following conditions—mucosal thickening, mucosal retention cyst, sinusitis, or foreign body/antrolith/tooth root—were detected in the MS, the MS was labeled “pathology present.” MSs without any of these pathologies were labeled “no pathology.” In total, 443 MSs were labeled “no pathology,” and 557 MSs were labeled “pathology present.” Semiautomatic software (ITK-SNAP) was used to manually generate the segmentation masks, and the outputs were compared against these manual segmentations (Fig. 1 (a), (b), (d), (e), (g), (h)). Initially, in semiautomatic segmentation of sinus pathologies, a custom threshold leveling was adjusted between [− 520 to − 52 Hounsfield units] to create a mask of the soft tissue. The boundaries of the sinus pathology were subsequently confirmed to be drawn correctly in all three sections (axial, coronal and sagittal). Missing/excessive parts were corrected manually. After the segmentation process, the data were converted to the Neuroimaging Informatics Technology Initiative (NIfTI) format to be transferred to the Dentmetria (Turkey) artificial intelligence program.

### Machine learning algorithm

A machine learning algorithm based on a CNN was used to segment maxillary sinus pathology from CBCT images (Fig. 1 (c), (f), (i)). The Dice similarity coefficient (DSC) and intersection over union (IoU) were used to measure the accuracy of segmentation when comparing the human-generated and AI-generated results.

### Model pipeline

The approach consisted of the following steps:

- Preprocessing the CBCT image.
- Classifying each voxel in the image as pathology or background.
- Reconstruct the pathology volumetric image.

### Semantic segmentation

The machine learning problem is expressed as semantic segmentation, which involves classifying each pixel in an image. The model classified each voxel as pathological or background. A U^2^-Net architecture was used for this task. U^2^-Net, an encoder-decoder neural network, captures semantic information through its encoding path and recovers spatial information via its decoding path.

### Implementation

The U^2^-Net architecture was implemented in Python. Training and experiments were conducted on an NVIDIA^®^ GeForce^®^ RTX 3090 GPU. The network was trained with the Adam optimizer. The loss function used was sparse categorical cross-entropy. The batch size was set to 16, with a learning rate of 0.0002. The network was trained for 40 epochs, and the model with the best validation loss was selected for testing.

### Statistical analysis

The performance of the segmentation procedure was assessed via the Dice similarity coefficient (DSC) and the intersection over union (IoU) metrics. The DSC was calculated to evaluate the segmentation performance, whereas the IoU was used to measure the pathology localization capabilities.

The segmentation was performed by the consensus of two experts in two dentomaxillofacial radiologists (A.K. & C.A.B.) with eight years of experience and subsequently reassessed by two senior radiologists (S.A. & K.O.) with at least 15 years of experience.

## Results

**Fig. 2:**
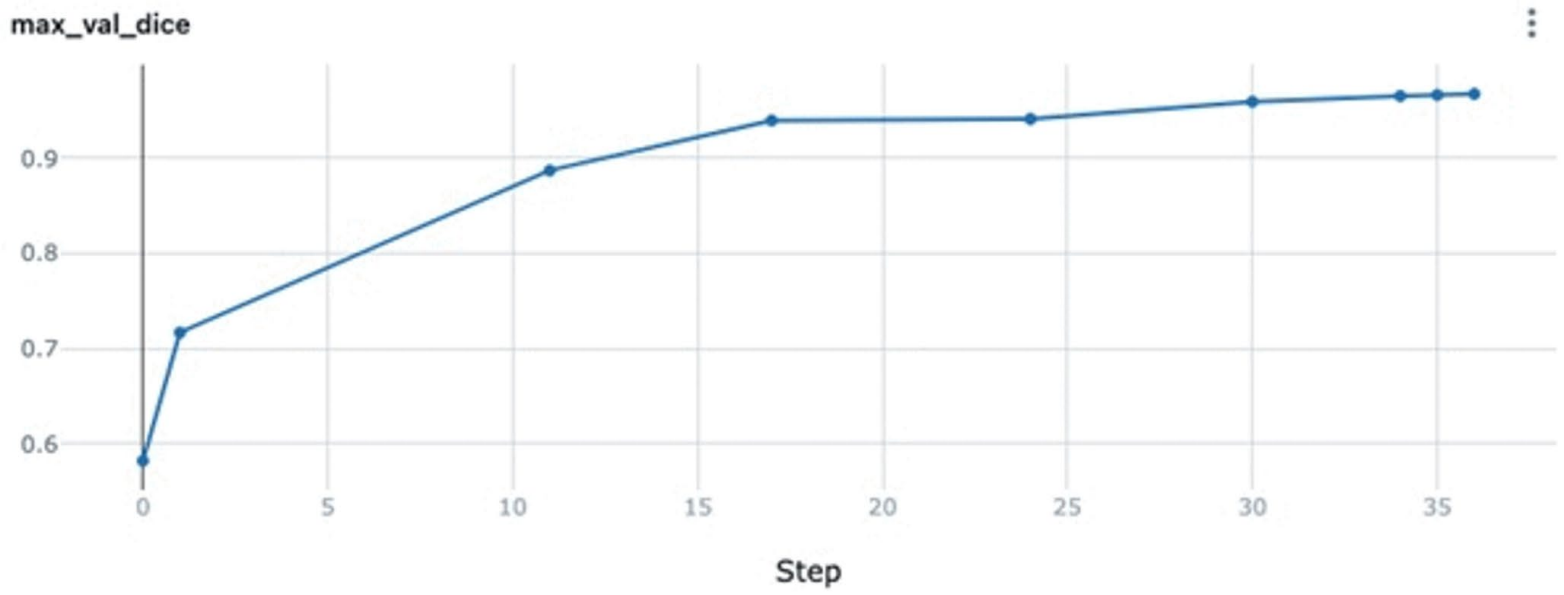
Step vs. max validation dice achieved

The automated segmentation model achieved acceptable performance in accurately segmenting MS pathologies from CBCT images. The model achieved a test DSC of 0.923, indicating a high degree of overlap between the AI-generated segmentation masks and the manual ground truth segmentations produced by expert radiologists. A DSC value closer to 1 signifies near-perfect agreement, underscoring the model’s ability to replicate human-level segmentation accuracy (Fig. 2).

**Fig. 3:**
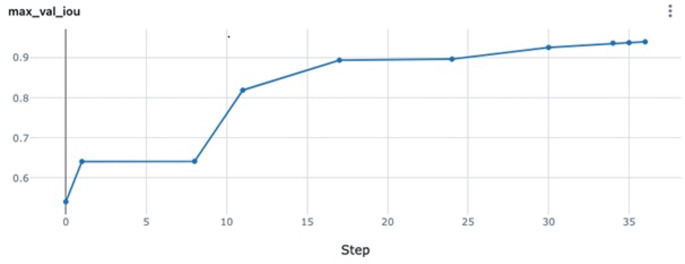
Step vs. max IoU achievements

The IoU score was 0.887, which shows the model’s precise localization capabilities in identifying the pathological regions within the MS. An IoU score approaching 1 denotes minimal discrepancy between the predicted and actual lesion areas, further confirming the model’s spatial accuracy in segmenting diverse MS pathologies (Fig. 3).

In terms of detection performance, the model attained a test recall (sensitivity) of 0.979. This high recall value indicates that 97.9% of the actual MS pathologies present in the test set were correctly identified by the algorithm, demonstrating its effectiveness in minimizing false negatives. The test precision (positive predictive value) was 0.963, revealing that 96.3% of the pathologies detected by the model corresponded to true positives. The balance between high recall and precision is encapsulated in the test F1 score of 0.970, which is the harmonic mean of precision and recall. This F1 score highlights the model’s overall robustness in both identifying true pathologies and avoiding incorrect detections (Fig. 3).

## Discussion

Recent advancements in CNNs have enabled automated learning of imaging characteristics, allowing the creation of predictive models. When test images are processed through a trained model, it generates an inference-based evaluation. This technique facilitates the classification of sinuses based on inflammation presence. However, unless an automated system is in place to isolate and crop the sinus region from the full image, manual intervention remains necessary. To enhance efficiency, these steps must be automated during both training and inference phases of AI-based segmentation [24, 25].

Model training involves processing numerous labeled images, where annotations indicate pathology types and sinus coordinates. This structured labeling aids in model creation. Once a trained model processes test images, it swiftly identifies and delineates the maxillary sinus, assigning different color-coded labels for each class during the inference phase [26].

Studies have demonstrated that deep learning algorithms effectively detect MS pathologies from two-dimensional radiographic images [24, 25]. However, CBCT provides a more detailed three-dimensional perspective, significantly improving diagnostic accuracy. Beyond simple detection, CBCT enhances evaluations of lesion morphology, volume, and spatial distribution, making it an optimal data source for AI-driven diagnostic tools in maxillofacial assessments [27].

CNN models are frequently employed for MS pathology detection in CT and CBCT scans. Literature findings suggest that AI models are generally effective in MS segmentation [2, 28]. Hung et al. [2] reported that 3D CNN models perform well in automatic detection, segmentation, and assessment of mucosal thickening and mucus retention cysts in CBCT scans. Their study indicated that CNN-based segmentation assists clinicians in identifying lesions, assessing sinus opacification, and planning surgical interventions. Similarly, Jung et al. [28] developed a CNN model tailored for CBCT-based MS pathology segmentation, successfully identifying mucosal thickening and mucus retention cysts. While their findings highlighted the model’s strong performance, challenges arose in distinguishing lesions due to variable radiopacity and soft tissue thickness variations in the sinus wall.

Among deep learning architectures, U-Net is widely recognized for its success in medical image segmentation. This model comprises an encoder that condenses input images into compact feature maps and a decoder that reconstructs them at their original scale. Its ability to enhance high-resolution image segmentation makes it a preferred choice for detecting anatomical structures, tumor localization, and lesion delineation [29]. U-Net’s efficiency in training on limited datasets, combined with rapid processing speeds, further supports its application in medical imaging.

In this study, a U^2^-Net-based CNN model was employed for MS segmentation. Prior research, such as that conducted by Bayrakdar et al. [7], demonstrated the effectiveness of U-Net-based CNN models for MS pathology segmentation, achieving high DC and IoU scores. Similarly, studies by Choi et al. [27] and Morgan et al. [30] reported DC scores ranging from 0.90 to 0.98 for U-Net-assisted segmentation. Consistent with these findings, our model achieved a segmentation accuracy of 0.923 and an MS pathology localization accuracy of 0.887, reinforcing the effectiveness of AI-driven methods for sinus pathology detection.

The integration of AI-based segmentation models in clinical practice has the potential to improve diagnostic accuracy and streamline workflows for dental professionals and radiologists. Automated segmentation can assist in early identification of sinus pathologies, thereby enhancing preoperative planning for dental implant placement and sinus-related surgical procedures. Additionally, AI-based segmentation can reduce the reliance on human expertise, leading to more consistent and reproducible evaluations, particularly in high-volume clinical settings.

Despite the promising results, certain limitations must be considered. The performance of AI models largely depends on the quality and diversity of the training dataset. Variability in CBCT scan settings, anatomical differences among patients, and image artifacts can impact model robustness. Additionally, while AI-driven segmentation reduces the time required for interpretation, manual verification by experts remains necessary to ensure clinical reliability. Future research should focus on enhancing dataset diversity and developing methods to improve AI generalizability across different imaging conditions.

Although U^2^-Net demonstrated strong segmentation performance, alternative deep learning models such as ResNet, V-Net, and Transformer-based architectures have also been explored for medical image analysis. A comparative analysis of these models could provide further insights into their strengths and weaknesses in MS pathology detection. Moreover, ensemble models that combine multiple AI architectures may enhance segmentation accuracy and robustness.

Future research should aim to integrate AI models into real-time CBCT scanning software to facilitate instant segmentation during imaging. Additionally, advancements in transfer learning and multimodal image analysis could further enhance AI performance by incorporating data from different imaging modalities, such as MRI and panoramic radiography. The application of AI beyond sinus pathologies, including detection of other maxillofacial conditions, could broaden its clinical utility and further support personalized patient care.

## Conclusions

The findings of this study confirm that deep learning algorithms, specifically those utilizing U^2^-Net convolutional neural network architectures, demonstrate reliable and precise segmentation of MS pathologies from CBCT scans. These results underscore the potential of AI-integrated diagnostic tools in maxillofacial radiology, aiding clinicians in the accurate and efficient evaluation of MS conditions. By addressing current limitations and exploring future advancements, AI-assisted technologies have the potential to revolutionize diagnostic imaging and treatment planning in dental and maxillofacial practice.

## Data Availability

All data generated or analyzed during this study are included in this published article.

## References

[1] Hung KF, Hui LL, Leung YY (2022) Patient-specific Estimation of the bone graft volume needed for maxillary sinus floor elevation: a radiographic study using cone-beam computed tomography. Clin Oral Investig 26:3875–3884. 10.1007/s00784-021-04354-0 35112191 10.1007/s00784-021-04354-0

[2] Hung KF, Ai QYH, King AD, Bornstein MM, Wong LM, Leung YY (2022) Automatic detection and segmentation of morphological changes of the maxillary sinus mucosa on cone-beam computed tomography images using a three-dimensional convolutional neural network. Clin Oral Investig 26:3987–3998. 10.1007/s00784-021-04365-x 35032193 10.1007/s00784-021-04365-x

[3] Yeung AWK, Colsoul N, Montalvao C, Hung K, Jacobs R, Bornstein MM (2019) Visibility, location, and morphology of the primary maxillary sinus ostium and presence of accessory Ostia: a retrospective analysis using cone beam computed tomography (CBCT). Clin Oral Investig 23(11):3977–3986. 10.1007/s00784-019-02829-9 30737619 10.1007/s00784-019-02829-9

[4] Bisla S, Gupta A, Singh H, Sehrawat A, Shukla S (2022) Evaluation of relationship between odontogenic infections and maxillary sinus changes: A cone beam computed Tomography-based study. J Oral Biol Craniofac Res 12:645–650. 10.1016/j.jobcr.2022.08.001 36045940 10.1016/j.jobcr.2022.08.001 PMC9421310

[5] Peñarrocha-Oltra S, Soto-Peñaloza D, Bagán-Debón L, Bagan JV, Peñarrocha-Oltra D (2020) Association between maxillary sinus pathology and odontogenic lesions in patients evaluated by cone beam computed tomography. A systematic review and meta-analysis. Med Oral Patol Oral Cir Bucal 25:e34–e48. 10.4317/medoral.23172 31880293 10.4317/medoral.23172 PMC6982991

[6] Çelebi A, Imak A, Üzen H, Budak Ü, Türkoğlu M, Hanbay D, Şengür A (2024) Maxillary sinus detection on cone beam computed tomography images using ResNet and Swin Transformer-based UNet. Oral Surg Oral Med Oral Pathol Oral Radiol 138:149–161. 10.1016/j.oooo.2023.06.001 37633787 10.1016/j.oooo.2023.06.001

[7] Bayrakdar IS, Elfayome NS, Hussien RA, Gulsen IT, Kuran A, Gunes I, Al-Badr A, Celik O, Orhan K (2024) Artificial intelligence system for automatic maxillary sinus segmentation on cone beam computed tomography images. Dentomaxillofac Radiol 53:256–266. 10.1093/dmfr/twae012 38502963 10.1093/dmfr/twae012 PMC11056744

[8] Schwendicke F, Samek W, Krois J (2020) Artificial intelligence in dentistry: chances and challenges. J Dent Res 99:769–774. 10.1177/0022034520915714 32315260 10.1177/0022034520915714 PMC7309354

[9] Hung KF, Ai QYH, Leung YY, Yeung AWK (2022) Potential and impact of artificial intelligence algorithms in dento-maxillofacial radiology. Clin Oral Investig 26:5535–5555. 10.1007/s00784-022-04477-y 35438326 10.1007/s00784-022-04477-y

[10] Leite AF, Vasconcelos KF, Willems H, Jacobs R (2020) Radiomics and machine learning in oral healthcare. Proteom Clin Appl 14:e1900040. 10.1002/prca.201900040 10.1002/prca.201900040 31950592

[11] Fu Y, Lei Y, Wang T, Curran WJ, Liu T, Yang X (2021) A review of deep learning based methods for medical image multi-organ segmentation. Phys Med 85:107–122. 10.1016/j.ejmp.2021.05.003 33992856 10.1016/j.ejmp.2021.05.003 PMC8217246

[12] Huang Y, Bert C, Sommer P, Frey B, Gaipl U, Distel LV, Weissmann T, Uder M, Schmidt MA, Dörfler A, Maier A, Fietkau R, Putz F (2022) Deep learning for brain metastasis detection and segmentation in longitudinal MRI data. Med Phys 49:5773–5786. 10.1002/mp.15863 35833351 10.1002/mp.15863

[13] Kijowski R, Liu F, Caliva F, Pedoia V (2020) Deep learning for lesion detection, progression, and prediction of musculoskeletal disease. J Magn Reson Imaging 52:1607–1619. 10.1002/jmri.27001 31763739 10.1002/jmri.27001 PMC7251925

[14] Kuntz S, Krieghoff-Henning E, Kather JN, Jutzi T, Höhn J, Kiehl L, Hekler A, Alwers E, von Kalle C, Fröhling S, Utikal JS, Brenner H, Hoffmeister M, Brinker TJ (2021) Gastrointestinal cancer classification and prognostication from histology using deep learning: systematic review. Eur J Cancer 155:200–215. 10.1016/j.ejca.2021.07.012 34391053 10.1016/j.ejca.2021.07.012

[15] Sukegawa S, Tanaka F, Nakano K, Hara T, Yoshii K, Yamashita K, Ono S, Takabatake K, Kawai H, Nagatsuka H, Furuki Y (2022) Effective deep learning for oral exfoliative cytology classification. Sci Rep 12:13281. 10.1038/s41598-022-17602-4 35918498 10.1038/s41598-022-17602-4 PMC9346110

[16] Schwendicke F, Chaurasia A, Arsiwala L, Lee JH, Elhennawy K, Jost-Brinkmann PG, Demarco F, Krois J (2021) Deep learning for cephalometric landmark detection: systematic review and meta-analysis. Clin Oral Investig 25:4299–4309. 10.1007/s00784-021-03990-w 34046742 10.1007/s00784-021-03990-w PMC8310492

[17] Sin Ç, Akkaya N, Aksoy S, Orhan K, Öz U (2021) A deep learning algorithm proposal to automatic pharyngeal airway detection and segmentation on CBCT images. Orthod Craniofac Res 24(Suppl 2):117–123. 10.1111/ocr.12480 33619828 10.1111/ocr.12480

[18] Järnstedt J, Sahlsten J, Jaskari J, Kaski K, Mehtonen H, Lin Z, Hietanen A, Sundqvist O, Varjonen V, Mattila V, Prapayasotok S, Nalampang S (2022) Comparison of deep learning segmentation and multigrader-annotated mandibular canals of multicenter CBCT scans. Sci Rep 12:18598. 10.1038/s41598-022-20605-w 36329051 10.1038/s41598-022-20605-w PMC9633839

[19] Son DM, Yoon YA, Kwon HJ, An CH, Lee SH (2021) Automatic detection of mandibular fractures in panoramic radiographs using deep learning. Diagnostics (Basel) 11:933. 10.3390/diagnostics11060933 34067462 10.3390/diagnostics11060933 PMC8224557

[20] Yilmaz E, Kayikcioglu T, Kayipmaz S (2017) Computer-aided diagnosis of periapical cyst and keratocystic odontogenic tumor on cone beam computed tomography. Comput Methods Programs Biomed 146:91–100. 10.1016/j.cmpb.2017.05.012 28688493 10.1016/j.cmpb.2017.05.012

[21] Amasya H, Alkhader M, Serindere G, Futyma-Gąbka K, Aktuna Belgin C, Gusarev M, Ezhov M, Różyło-Kalinowska I, Önder M, Sanders A, Costa ALF, Castro Lopes SLP, Orhan K (2023) Evaluation of a decision support system developed with deep learning approach for detecting dental caries with cone-beam computed tomography imaging. Diagnostics (Basel) 13:3471. 10.3390/diagnostics13223471 37998607 10.3390/diagnostics13223471 PMC10669958

[22] Orhan K, Aktuna Belgin C, Manulis D, Golitsyna M, Bayrak S, Aksoy S, Sanders A, Önder M, Ezhov M, Shamshiev M, Gusarev M, Shlenskii V (2023) Determining the reliability of diagnosis and treatment using artificial intelligence software with panoramic radiographs. Imaging Sci Dent 53:199–208. 10.5624/isd.20230109 37799743 10.5624/isd.20230109 PMC10548159

[23] Schwendicke F, Singh T, Lee JH, Gaudin R, Chaurasia A, Wiegand T, Uribe S, Krois J (2021) Artificial intelligence in dental research: checklist for authors, reviewers, readers. J Dent 107:103610. 10.1016/j.jdent.2021.103610

[24] Kuwana R, Ariji Y, Fukuda M, Kise Y, Nozawa M, Kuwada C, Muramatsu C, Katsumata A, Fujita H, Ariji E (2021) Performance of deep learning object detection technology in the detection and diagnosis of maxillary sinus lesions on panoramic radiographs. Dentomaxillofac Radiol 50:20200171. 10.1259/dmfr.20200171 32618480 10.1259/dmfr.20200171 PMC7780831

[25] Hiraiwa T, Ariji Y, Fukuda M, Kise Y, Nakata K, Katsumata A, Fujita H, Ariji E (2019) A deep-learning artificial intelligence system for assessment of root morphology of the mandibular first molar on panoramic radiography. Dentomaxillofac Radiol 48:20180218. 10.1259/dmfr.20180218 30379570 10.1259/dmfr.20180218 PMC6476355

[26] Ariji Y, Yanashita Y, Kutsuna S, Muramatsu C, Fukuda M, Kise Y, Nozawa M, Kuwada C, Fujita H, Katsumata A, Ariji E (2019) Automatic detection and classification of radiolucent lesions in the mandible on panoramic radiographs using a deep learning object detection technique. Oral Surg Oral Med Oral Pathol Oral Radiol 128:424–430. 10.1016/j.oooo.2019.05.014 31320299 10.1016/j.oooo.2019.05.014

[27] Choi H, Jeon KJ, Kim YH, Ha EG, Lee C, Han SS (2022) Deep learning-based fully automatic segmentation of the maxillary sinus on cone-beam computed tomographic images. Sci Rep 12:14009. 10.1038/s41598-022-18436-w 35978086 10.1038/s41598-022-18436-w PMC9385721

[28] Jung SK, Lim HK, Lee S, Cho Y, Song IS (2021) Deep active learning for automatic segmentation of maxillary sinus lesions using a convolutional neural network. Diagnostics (Basel) 11:688. 10.3390/diagnostics11040688 33921353 10.3390/diagnostics11040688 PMC8070431

[29] Ozturk B, Taspinar YS, Koklu M, Tassoker M (2024) Automatic segmentation of the maxillary sinus on cone beam computed tomographic images with U-Net deep learning model. Eur Arch Otorhinolaryngol 281:6111–6121. 10.1007/s00405-024-08870-z 39083060 10.1007/s00405-024-08870-z PMC11512868

[30] Morgan N, Van Gerven A, Smolders A, de Faria Vasconcelos K, Willems H, Jacobs R (2022) Convolutional neural network for automatic maxillary sinus segmentation on cone-beam computed tomographic images. Sci Rep 12:7523. 10.1038/s41598-022-11483-3 35525857 10.1038/s41598-022-11483-3 PMC9079060

